# Access to a dementia-friendly garden on behavioural and psychological symptoms of dementia, falls and psychotropic medication use in residents of an aged care home in Melbourne, Australia

**DOI:** 10.1177/10398562231160363

**Published:** 2023-03-02

**Authors:** Rhoda Lai, Mouhamed Foladkar, Gurnik Dhaliwal, Anika Kibria, Rosa C Gualano, Madeleine L Healy

**Affiliations:** 589637 Monash Ageing Research Centre (MONARC), Department of Medicine, Monash University, Melbourne, VIC, Australia; Rehabilitation and Aged Care Services, 2538 Monash Health, Melbourne, VIC, Australia; Rehabilitation and Aged Care Services, 2538 Monash Health, Melbourne, VIC, Australia; 589637 Monash Ageing Research Centre (MONARC), Department of Medicine, Monash University, Melbourne, VIC, Australia;; Rehabilitation and Aged Care Services, 2538 Monash Health, Melbourne, VIC, Australia; 589637 Monash Ageing Research Centre (MONARC), Department of Medicine, Monash University, Melbourne, VIC, Australia;; Rehabilitation and Aged Care Services, 2538 Monash Health, Melbourne, VIC, Australia; 589637 Monash Ageing Research Centre (MONARC), Department of Medicine, Monash University, Melbourne, VIC, Australia;; Rehabilitation and Aged Care Services, 2538 Monash Health, Melbourne, VIC, Australia, Dementia Support Australia

**Keywords:** Dementia, nursing homes, Australia, gardens

## Abstract

**Objective:**

Residents of care homes need access to outdoors. This may improve behavioural and psychological symptoms of dementia (BPSD) and quality of life in residents living with dementia. Barriers including lack of accessibility and increased falls risk, which may be mitigated using dementia-friendly design. This prospective cohort study followed a group of residents in the first 6 months after the opening of a new dementia-friendly garden.

**Method:**

Nineteen residents participated. The Neuropsychiatric Inventory – Nursing Home Version (NPI-NH) and psychotropic medication use were collected at baseline, 3 and 6 months. The facility's falls rate during this time and feedback from staff and residents' next of kin were collected.

**Results:**

Total NPI-NH scores decreased, though not significantly. Feedback was positive overall; the falls rate decreased. Usage of the garden was low.

**Conclusions:**

Despite its limitations, this pilot study adds to the literature about the importance of access to the outdoors for people who are experiencing BPSD. Staff remain concerned about falls risk despite the dementia-friendly design, and many residents do not access outdoors frequently. Further education may help to remove barriers to encouraging residents to access the outdoors.

Behavioural and psychological symptoms of dementia (BPSD) are common, in up to 95% of people living with dementia.^2^ BPSD can be due to external factors such as the environment or internal factors such as pain or depression. Management must be personalised and primarily non-pharmacological.^3^ Unfortunately, both in Australia and internationally, psychotropic medication use remains common.^4,5^

Non-pharmacological management includes unrestricted access to the environment.^6^ The Aged Care Quality and Safety Commission’s fifth standard requires that the residential aged care facility (RACF) *‘helps consumers to move freely in the environment (including access to outdoor areas)’*.^7^ Barriers include lack of accessibility and perceived falls risk.^8^ Dementia design could mitigate these barriers.^9^

Access to the outdoors presumably improves symptoms of BPSD by addressing unmet needs, but evidence is limited. A current focus of dementia-friendly design is to address staff concerns about falls risk and to mitigate barriers to outdoor access.^8^ Previous studies have documented reduction in behaviours from time spent outdoors in dementia-friendly outdoor environments^6,10-12^ and improvement in quality of life,^11,13^ depression^13,14^ and sleep.^13^ However, these were all small studies from a variety of settings. There are very limited data from small observational studies on garden use and potential for reductions in medication.^9^

## Aims

This prospective cohort study evaluated usage of a dementia-friendly outdoor space by aged care residents living with dementia as well as their BPSD severity, psychotropic medication use and falls rates during a 6-month period. It aimed to analyse data for trends or changes in these domains after establishment of the garden. It also aimed to record qualitative feedback from family, friends and staff on the use of the garden, its benefits, and any concerns to document barriers and enablers to garden usage.

## Method

### Setting and participants

The setting was a 39-bed RACF in a metropolitan health service in Melbourne, Victoria. Around 80% of residents had a diagnosis of dementia and were estimated to occupy tier 5–6 on the Brodaty triangle of severity.^15^ All had access to the garden regardless of participation in the study. Residents with BPSD were identified by staff, who requested permission from their next of kin (NOK) to be referred to the study team. NOK were provided with verbal and written information and provided written consent.^16^ Data were collected from September 2020 to April 2021.

### Data collection

Residents in the study were followed for 6 months with presence and severity of BPSD measured using the Neuropsychiatric Inventory – Nursing Home Version (NPI-NH)^17^ at baseline, 3 and 6 months. 3-monthly completion of the NPI-NH was part of standard care at the facility. The same staff member completed the NPI-NH for a participant at each time for consistency. The NPI-NH was used as it addresses aspects of BPSD beyond agitation, such as mental health (depression, anxiety), apathy, sleep and appetite, rather than the Cohen-Mansfield Agitation Inventory^18^ used by previous studies.^6,19,20^

Demographic data, diagnoses and antipsychotic, benzodiazepine and opiate use were taken from medical files. Falls/incident data were taken for the entire facility from the service’s reporting system, and were unidentifiable.

Feedback questionnaires were completed at 3 and 6 months by NOK and at 6 months by staff members.

### Data analysis

Descriptive analyses were used for demographics, garden use rates, falls rates and staff and family questionnaires. Due to the small sample size, a non-parametric Friedman test was used to analyse differences in NPI-NH total scores over the three time points. Wilcoxon signed-rank tests were used to analyse differences between baseline and the 3- and 6-month time points in regular and PRN psychotropic medication use of each type. Due to the small sample size, qualitative analysis was not formally undertaken but all comments have been represented in the results.

## Results

### Demographics and garden use

Nineteen residents (9 female, 47%; 10 male, 53%) were included in the study. The mean age was 71 years, and they had lived at the RACF for an average of 41 months at baseline. Nine were diagnosed with Alzheimer’s disease, three with vascular dementia, five with dementia unspecified and one each with frontotemporal and mixed dementia types. See Table 1 for comorbidities. Two participants were deceased by the 3-month follow-up, and two more by 6 months.

**Table 1.** Participants’ comorbid diagnoses

Diagnosis	Number of participants
Depression and/or anxiety/PTSD	7
Diabetes mellitus	4
Schizophrenia	4
Congestive cardiac failure	3
Epilepsy	3
Pain	3
ABI or other cognitive impairment	2
Arthritis (osteoarthritis or inflammatory)	2
Hearing and/or visual impairment	2
Ischaemic heart disease	2
Malignancy	2
Stroke	2
Abdominal aortic aneurysm	1
Atrial fibrillation	1
Chronic kidney disease	1
Chronic liver disease	1
Chronic obstructive pulmonary disease	1
Gastro-oesophageal reflux disease	1
Joint replacement (hip)	1
Osteoporosis/fracture	1
Average number of comorbidities (per participant)	3

Usage of the garden was low, with participants using the garden an average of 1.47 times per week. Overall, they went outdoors at least weekly 56% of the time, and at least three times a week 31% of the time. One participant never used the garden, and one more used the garden only once. Weather (including rain, cold and heat) was noted as a barrier in 6 weeks. In retrospect it was recognised that residents could see the garden and enjoy it from inside communal areas and rooms, but this was not measured formally.

### NPI-NH data

A Friedman test was run to determine if there were differences in NPI-NH total scores over 6 months of access to the garden space. NPI-NH scores decreased from baseline (Median = 34) to 3 months (Median = 18), then slightly increased at 6 months (Median = 20), but the differences were not statistically significant, χ2(2) = 2.07, *p* = 0.4. Participants who did not survive to 6-month follow-up were not included in the Friedman test, which did not change the overall trend.

**Figure 1.**
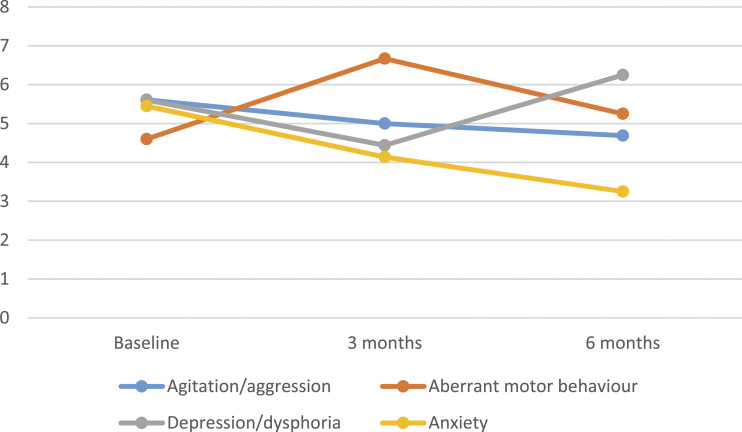
Changes in frequency x severity scores of selected NPI-NH sub-sections over time. *Note.* NPI-NH = Neuropsychiatric Inventory Nursing Home Version.

**Figure 2.**
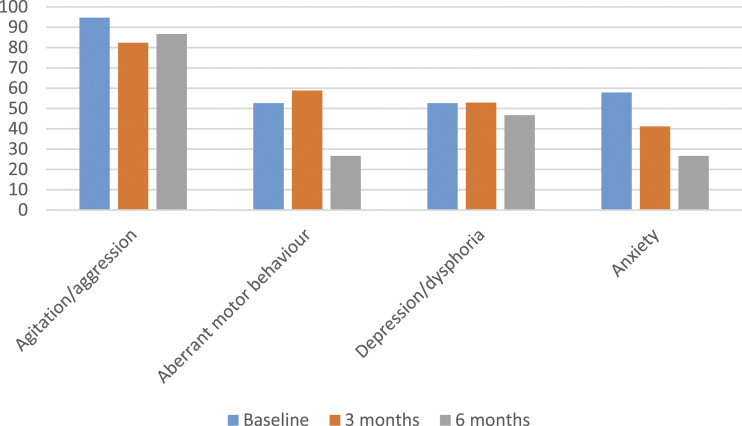
Percentage of participants with selected BPSD as recorded on the NPI-NH over time. *Note.* BPSD = behavioural and psychological symptoms of dementia; NPI-NH = Neuropsychiatric Inventory Nursing Home Version.

Trends in the ‘agitation/aggression’, ‘aberrant motor behaviour’, ‘depression/dysphoria’ and ‘anxiety’ sub-sections of the NPI-NH were hypothesised to be most impacted by access to the garden, based on current literature. Some positive trends can be observed in Figures 1 and 2, though meaningful subscale analysis is not reliable given the small sample size.

### Medication use and falls data

Wilcoxon signed-rank tests indicated no significant differences in regular or PRN medication use of either type between baseline and the 6 months of follow-up, except in PRN benzodiazepine use between baseline and 3 months, which significantly increased (*Z* = 15.00, *p* = 0.04). There was also a trend towards increasing regular benzodiazepine use and decreasing PRN antipsychotic use over the 6 months.

Over the study period, there were 29 falls. Over the same dates in the two years prior, 51 and 49 falls were recorded.

### Staff and family questionnaires

Eight staff completed questionnaires for 16 participants (Table 2). The most frequently listed barriers were weather and the need for assistance to access the garden (eight participants), with one staff member mentioning a lack of carer help due to the COVID-19 lockdowns. Other barriers included lack of interest in going outside and safety concerns. The most frequent suggestions (11 participants) were flattening surfaces/slopes and removing large rocks, to make it easier to wheel residents in chairs and mitigate concerns about trips/falls (eight participants). Other suggestions included adding more colour, sensory material, painted guide markers around the edge of the footpath and a larger common area.

**Table 2.** Staff questionnaire responses

Question and response options		Number	Percentage
*Time spent outside before garden was built*			
	Never	2	12
	Rarely	7	44
	Some days	3	19
	Most days	3	19
	Every day	1	6
*Time spent outside in new garden*			
	Never	2	12
	Rarely	2	13
	Some days	8	50
	Most days	3	19
	Every day	1	6
*Activities (choose all that apply)*			
	Walking	7	44
	Sitting outdoors	8	50
	Spending time with visitors	3	19
	Activities, for example, reading/music	2	13
	Gardening/observing plants	1	6
	Other	2	13
*Benefit for agitation/distress*			
	Not at all	2	13
	Sometimes	4	25
	More often than not	5	31
	Always	1	6
	Major benefit	3	19
	Not applicable	1	6
Total surveys		16	—

Eight questionnaires were returned from NOK (four at each time point; see Table 3). An additional five NOKs provided only qualitative feedback (three at 3 months and two at six months). Feedback indicated that needing assistance to access the garden was a barrier, but no concerns about risks were listed by any NOKs, except one who noted that other residents could be affected by their relative moving slowly. Several noted that their relative enjoyed the garden, including from looking out the window. While a few stated that their relative was not interested in going outside, they acknowledged that the garden was positive overall. Both staff and NOKs suggested adding more sensory material (e.g. colourful and textured plants or a bird feeder).

**Table 3.** Family questionnaire responses

Question and response options		Number	Percentage
*Before moving into residential care, how often did your relative like to spend time outdoors?*			
	Never	0	0
	Rarely	0	0
	Some days	2	25
	Most days	5	63
	Every day	1	12
*How often has your relative been spending time in the sensory garden at the care home?*			
	Never	0	0
	Rarely	2	25
	Some days	3	38
	Most days	1	12
	Every day	0	0
	Unknown	2	25
*Which of the following activities does your relative take part in outdoors? (Tick all that apply)*			
	Walking	3	38
	Sitting outdoors	6	75
	Spending time with visitors	3	38
	Activities, for example, reading/music	1	13
	Gardening/observing plants	1	13
	Other	0	0
*Are you satisfied with the amount of time your relative is spending in the sensory garden?*			
	Yes	3	38
	Would like more time	5	62
	Would like less time	0	0
*Are you satisfied with the access hours (7am–7pm) to the sensory garden?*			
	Yes	8	100
	Would like longer	0	0
	Would like shorter	0	0
*How often have you had the opportunity to visit your relative at the care home on average in the last 3 months?*			
	Daily	0	0
	Weekly	5	62
	Monthly	3	38
	Less than monthly	0	0
Total surveys		8	—

## Discussion

This study adds to the current literature that access to outdoors is important for people experiencing BPSD in RACFs. There was a non-significant reduction in falls, overall symptoms and in the agitation/aggression and anxiety sub-scales for residents in this study. However, the study was limited by small numbers in a single RACF, and by infrequent garden use. The inherent day-to-day variability of BPSD is a potential limitation that could be less significant in a larger scale future evaluation.

There was no decrease in psychotropic medication use despite a previous study observing a decrease in the use of PRN medications.^6^ Although PRN antipsychotic use decreased, benzodiazepine use increased. The reasons for this are unclear, but of note, this study fell within a period of strict COVID-19 lockdown, when no family, friends or external providers visited, which may have affected BPSD.

It is important to consider why residents’ access to outdoors was so infrequent. BPSD management impacts on staff time^21^ so their perceptions of the garden’s impact and encouragement of its use are important. For 89% of residents, staff reported that time outdoors had some benefit for their agitation or distress, but despite this and the dementia-friendly design, staff were still concerned about falls.

While the risk of falls is often cited as a reason to prevent access to the outdoors,^8^ there is limited supportive literature. Falls are common in older adults, and dementia^22^ and frequent use of psychotropic medication increase risk.^21^ Falls can lead to morbidity and mortality,^22^ so the concerns of staff are valid. Notwithstanding, falls rates decreased in our study period compared to the previous 2 years. Similarly, Detweiler et al.^6^ observed a 30% decreased falls rate in a group of residents who were common users of a dementia ‘wander garden’, suggesting that outdoor access may actually reduce falls risk. Education for aged care staff could lead to improved use of outdoor spaces. Despite this limitation, the garden enhanced the indoor environment and could also be enjoyed through windows, which may have also impacted on BPSD and mood, and this warrants exploration in future studies.

Responses from family were limited (Table 3) as the COVID-19 lockdown during the study period made it difficult for NOKs to complete the questionnaires reliably when not visiting. However, the replies from family were positive and feedback was centred around features that could add enjoyment, rather than safety concerns.

## Conclusions

Access to the outdoors and the natural world is a fundamental right for all people, including people living with dementia. This prospective cohort study showed a non-significant trend towards decreasing agitation and falls. Actual usage of the garden was low. We noted staff concern about falls but positive feedback from families about benefits of the garden.
